# The application of biomaterials in osteogenesis: A bibliometric and visualized analysis

**DOI:** 10.3389/fbioe.2022.998257

**Published:** 2022-09-09

**Authors:** Jie Wang, Yuan Chi, Baohui Yang, Qiongchi Zhang, Dong Wang, Xijing He, Haopeng Li

**Affiliations:** ^1^ Department of Orthopedic Surgery, Second Affiliated Hospital of Xi’an Jiaotong University, Xi’an, China; ^2^ School of Medicine, Nankai University, Tianjin, China; ^3^ Department of Plastic and Reconstructive Surgery, The First Medical Centre, Chinese PLA General Hospital, Beijing, China

**Keywords:** biomaterials, osteogenesis, bibliometric analysis, orthopedic implants, osseointegration

## Abstract

Osteogenesis serves an important role in bone tissue repairing. Novel biomaterials are widely prevalent as materials for orthopedic implants due to their biocompatibility and osteogenetic ability. The purpose of this study was to comprehensively analyze hotspots and future trend of biomaterials research in osteogenesis based on bibliometric and visualized analysis. A total of 1,523 papers about biomaterials research in osteogenesis between 2000 and 2021 were included in this study. During the above 20 years, China’s leading position in the global biomaterials research in osteogenesis was obvious, and it was also the country that most frequently participates in international cooperation. Chinese Academy of Sciences was the most productive institution and the leader of research cooperation. Acta Biomaterialia and Biomaterials have published the largest number of articles in the field of biomaterials research in osteogenesis. Meanwhile, Acta Biomaterialia and Biomaterials were also the two journals with the highest total citation frequency. Wu CT, Chang J, Kaplan DL, and Xiao Y all made important contributions in the field of biomaterials research in osteogenesis. At present, there are five research hotspots in the field of biomaterials research in osteogenesis: 1) the immunomodulatory role of biomaterial-related inflammatory; 2) mechanisms of osteogenesis in biomaterials; 3) 3D printing and clinical application of biomaterials; 4) bone tissue engineering for biomaterial osteogenesis; and 5) regenerative medicine for biomaterial osteogenesis. The results of this study showed that mechanisms of osteogenesis in biomaterials, bone tissue engineering for biomaterial osteogenesis, and regenerative medicine for biomaterial osteogenesis will remain research hotspots in the future. International cooperation was also expected to expand and deepen the field of biomaterials research in osteogenesis.

## Introduction

Biomaterials have been widely used in fields of orthopedic implants over the past few decades, as injuries, joint and spinal diseases have increased and materials processing has improved (Lin et al., 2017; Kaur and Singh, 2019). The primary role of orthopedic implants, in the beginning, was to replace the bone tissue defects or maintain the morphology of bone tissue and bear certain stress effects (Navarro et al., 2008; Van Der Stok et al., 2011). Therefore, scholars, at that time, did not pay enough attention to the research on the growth and self-repair of bone tissue surrounding implants after implantation. With the development of bone tissue engineering and material science, the osseointegration between orthopedic implants and bone has attracted more and more attention. Osseointegration was defined as the process of achieving and maintaining rigid fixation between bone and implants in direct contact with the implants under a functional load (Albrektsson et al., 1981; Albrektsson and Johansson, 2001). However, Ti6Al4V widely used in clinical practice has poor osseointegration performance, which limits its early biological fixation and long-term stability with bone (Man et al., 2018; Harb et al., 2020; Avila et al., 2021). The reason for the above phenomenon is that Ti6Al4V is a biologically inert material, which has no bioactivity and osteoinductivity (Wu et al., 2008; Li et al., 2016a). The premise and key to achieve good osseointegration of orthopedic implants and bone is that the biomaterials used should have the ability to promote osteogenesis. Therefore, the development of novel biomaterials with osteogenic ability becomes a solution to improve the osseointegration performance of orthopedic implants.

At present, there are various methods to prepare novel biomaterials with osteogenic ability. It includes research and development of novel biomaterials, surface modification of biomaterials, and design of novel structures of biomaterials. The research and development of novel biomaterials included novel β titanium alloys, magnesium alloys, bioceramics, and polymers, etc., (Abdullah et al., 2015; Meischel et al., 2017; Furko et al., 2019; Li et al., 2019). Major surface modification techniques of biomaterials included sandblasting, acid-etching, de-alloying, and micro-arc oxidation, etc (Chou et al., 2017; Okulov et al., 2020; Zhang et al., 2021). Novel structural design of biomaterials included porous architecture design, individualized three-dimensional (3D) printing, and biomimetic design, etc (Wong, 2016; Yuan et al., 2019; Chen et al., 2021). However, at present, there is still a lack of systematic, intuitive, and visualized analysis to help researchers analyze the research trends in the field of biomaterials research in osteogenesis.

Bibliometric analysis is a novel science to analyze the contributions of countries or regions, institutions, authors and journals to a certain research field (Yin et al., 2021; Yin M. C. et al., 2022). In addition, bibliometric analysis can predict research hotspots and trends in a certain research field through information visualization (Cooper, 2015; Deng et al., 2022). However, bibliometric analysis has rarely been applied to the field of biomaterials research in osteogenesis.

In this study, we conducted a systematic bibliometric analysis of the research literature on biomaterials research in osteogenesis from 2000 to 2021, including the number of annual publications, countries or regions, international collaboration, institutions, journals, authors, and co-occurrence visualization analysis of keywords. In addition, the recent research advances in the field of biomaterials research in osteogenesis in the past 20 years were also prospected. At the same time, the research hotspots and trends of biomaterials research in osteogenesis were determined by using co-occurrence overlay visualization maps of keywords and double-clustering analysis. We hope that this study can provide a research basis and a new Frontier for the future biomaterials research in osteogenesis.

## Materials and methods

### Data sources and search strategy

The Web of Science database includes a large number of authoritative and high-impact academic journals. The data of this study were all from the Web of Science Core Collection on the website of Xi’an Jiaotong University Library. The retrieval strategy was TS= (Biomaterials AND osteogenesis).

### Screening criteria and data downloads

The publication dates of this study were searched from 2000 to 2021. Non-article, non-review, and non-English language publications are excluded. The selected data of the Web of Science Core Collection included titles, publication year, authorship, abstracts, keywords, source journals, organizations, countries or regions, and references, etc. The above data were downloaded in txt format. To avoid deviations caused by frequent database updates, all literature searches and data downloads were conducted on the same day (12 May 2022). Two scholars (JW and YC) conducted the retrieval independently. There was no statistical difference between the two groups, indicating consistency.

### Statistical analysis

This study systematically described the various characteristics of publications, including authors, institutions, countries or regions, journals, keywords, impact factor (IF), and Hirsch index (h-index). The IF derived from the Journal Citation Report (JCR) 2020 was used to assess the academic merit of research. Data extracted from the Web of Science Core Collection were imported into the online bibliometric analysis platform (http://bibliometric.com/) and VOSviewer Version 1.6.18 (Leiden University, Leiden, Netherlands) for bibliometric analysis. Apache ECharts is a Java language based data visualization tool for the visualized analysis of the number of annual publications and cumulative publications in different countries or regions. Online bibliometric analysis platform was used for visualized analysis of international cooperation between different countries or regions. The visual analysis of international cooperation in different countries or regions is helpful to evaluate and analyze the current trend of cooperation. VOSviewer was used to analyze and visualize bibliometric networks, including authors, institutions, journals, co-citations, and keywords. The analysis of these data was crucial to understanding the trends of popular journals, authors, and institutions. In addition, the analysis of keywords was helpful to systematically evaluate the current research hotspots and trends in the field of biomaterial osteogenesis. This also played a certain enlightenment role for the future development of this field. VOSviewer was used to generate network visualization maps and overlay visualization maps, which was helpful to intuitively grasp the trends in the field of biomaterial osteogenesis. Online bibliometric analysis platform and Microsoft Excel 2016 were used to evaluate the academic influence of authors, institutions, and journals. The data extracted from the Web of Science Core Collection were imported into the Bibliographic Item Co-occurrence Matrix Builder (BICOMB) to construct the keywords-articles binary matrix. The rows of the matrix represented publications and the columns represented high frequency keywords. Gcluto 1.0 software was used for double-clustering analysis, and matrix graph and volcano graph were constructed according to the results of the clustering analysis. The matrix diagram and volcano diagram visually showed the research hotspots and trends in the field of biomaterial osteogenesis.

## Results

### Trends and annual publications

**FIGURE 1:**
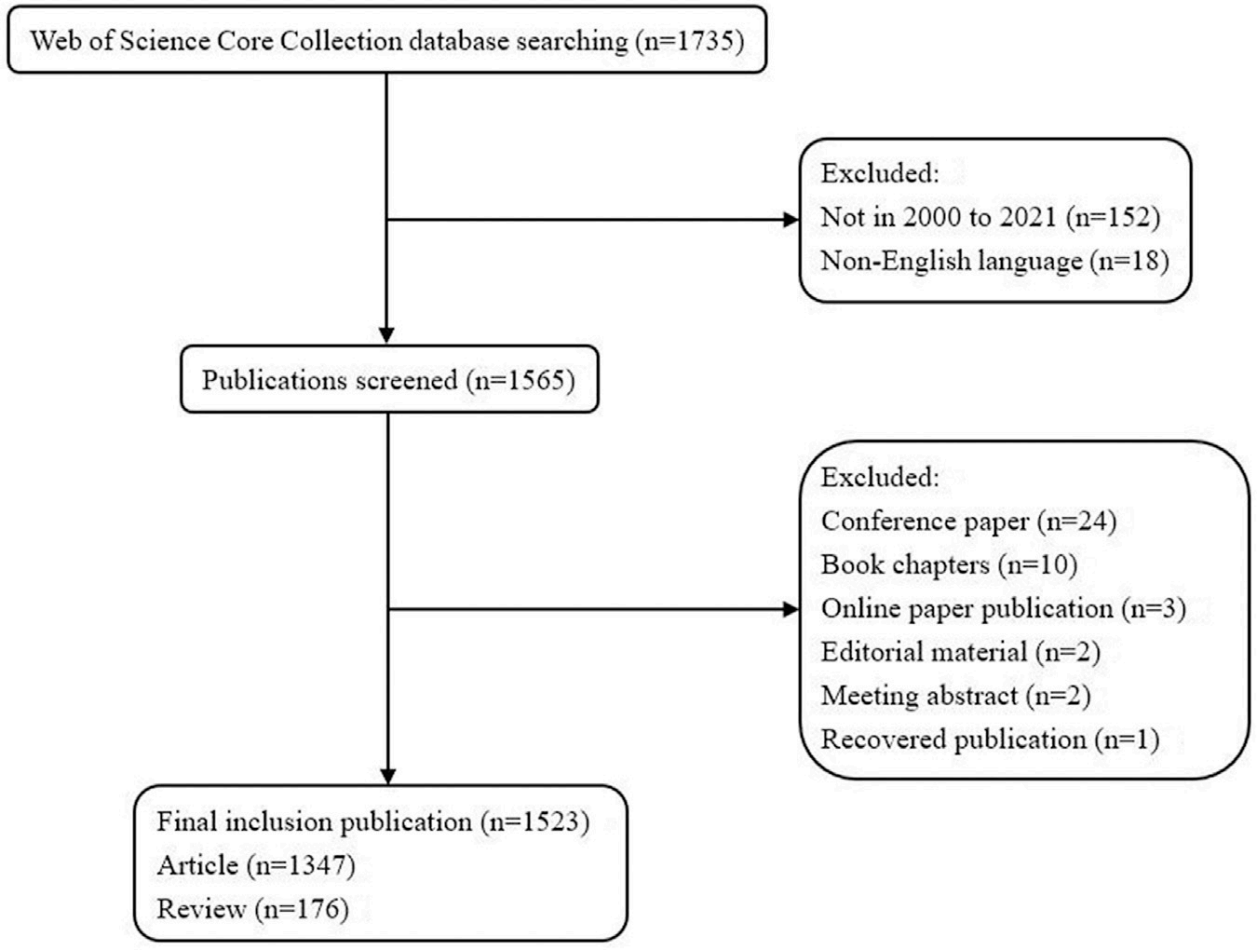
Flowchart of data screening and excluding.

**FIGURE 2:**
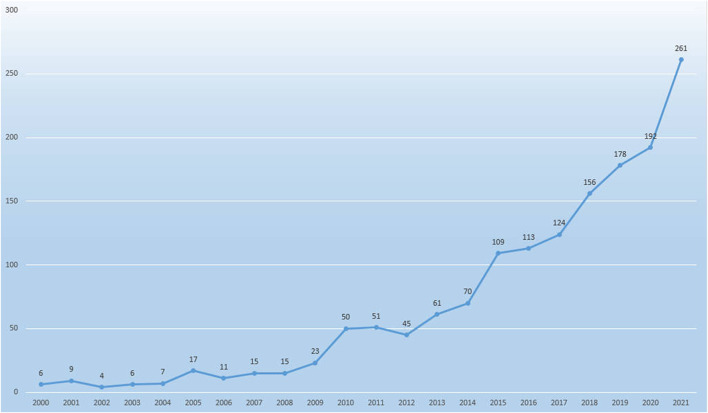
Annual number of the published publication in biomaterials research in osteogenesis from 2000 to 2021.

The process of data screening and excluding was shown in Figure 1. A total of 1735 papers were identified. According to the screening criteria, 1,523 papers (1,347 articles and 176 reviews) were included in this study. Figure 2 shows the gradual growth trend of annual publications related to biomaterials research in osteogenesis, from 6 papers in 2000 to 261 papers in 2021. Based on the Web of Science Core Collection database, 1,523 papers were cited 166,438 times, and each paper was cited 109.28 times on average.

### Contribution of countries and institutions

**FIGURE 3:**
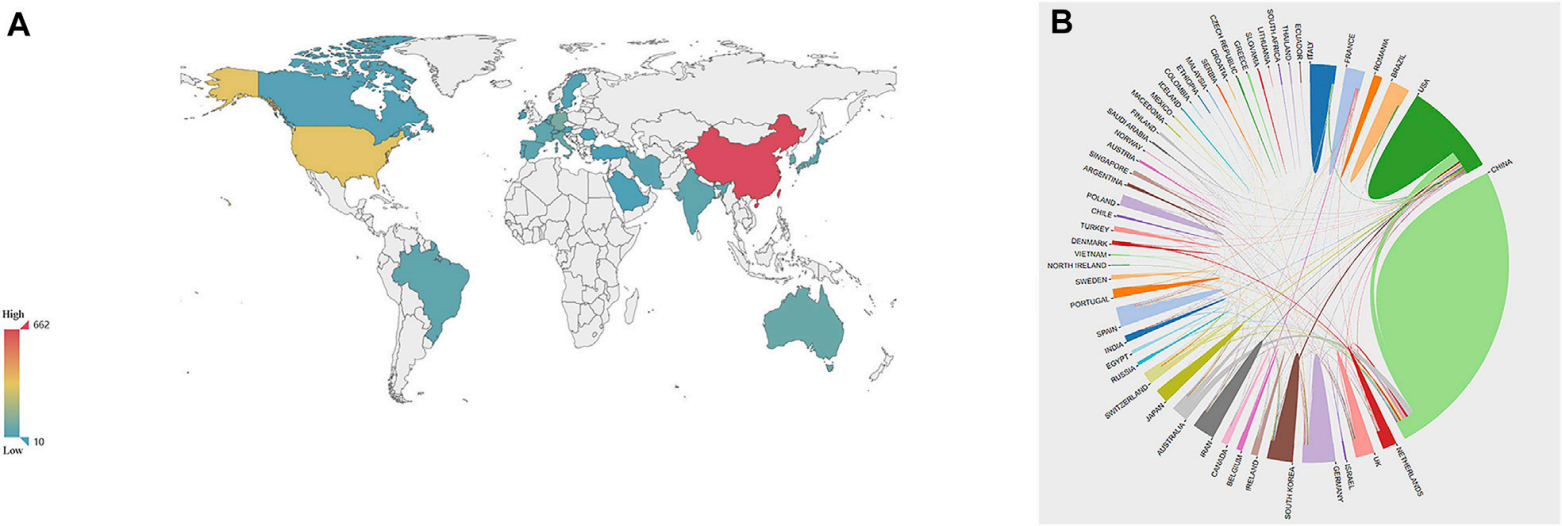
The distribution of countries or regions in biomaterials research in osteogenesis. **(A)** Distribution of biomaterials research in osteogenesis literatures in the world map. The color of each country or region on the world map represents the amount of literature published, according to the color gradient in the lower left corner. **(B)** The network map of cooperation between countries or regions. Different colors represent different countries or regions, the area of each color represents the amount of literature published in each country or region, and the thickness of the connecting line indicates the cooperation frequency.

According to the Web of Science Core Collection database, 64 countries or regions published publications on biomaterials research in osteogenesis between 2000 and 2021. The top 29 countries or regions in terms of the number of publications about biomaterials research in osteogenesis (n ≥ 10) were shown in the world map in Figure 3A, and the top 10 countries or regions were shown in Table 1.

**TABLE 1:** The top 10 countries or regions of publications in biomaterials research in osteogenesis.

Rank	Country/Region	Records	Percentage (*n*/1,523) %
1	China	662	43.467
2	United States	322	21.142
3	Germany	98	6.435
4	England	72	4.728
5	Italy	72	4.728
6	South Korea	72	4.728
7	Australia	69	4.531
8	Netherlands	50	3.283
9	Brazil	49	3.217
10	Japan	49	3.217

China published the most papers with 662, followed by the United States (*n* = 322), Germany (*n* = 98), England (*n* = 72), Italy (*n* = 72), South Korea (*n* = 72), Australia (*n* = 69), Netherlands (*n* = 50), Brazil (*n* = 49), and Japan (*n* = 49). The contribution of China and the United States to the number of published papers of biomaterials research in osteogenesis far exceeded that of other countries or regions (Table 1 and Figure 3A). Close cooperation between countries or regions of the world was extremely common during the investigation period. The analysis results of international cooperation showed that China was the country with the highest frequency of participating in international cooperation (Figure 3B).

**FIGURE 4:**
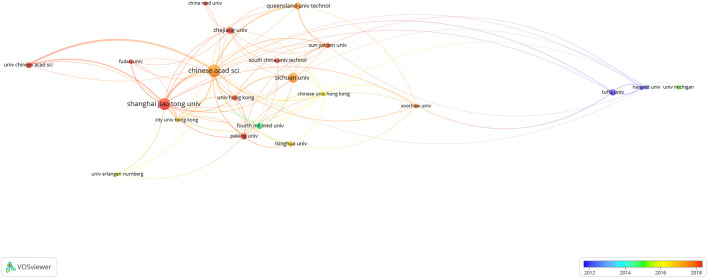
Co-authorship overlay visualization map of institutions. The color of each circle corresponds to the average publication year, the size of a circle is proportional to the number of literatures, and the thickness of the connecting line indicates the cooperation frequency.

Our study also assessed the most productive institutions for research. As shown in Table 2, The institution that published the most papers was Chinese Academy of Sciences, which published 125 papers. Followed by Shanghai Jiaotong University (*n* = 110), Sichuan University (*n* = 75), Zhejiang University (*n* = 37), Queensland University of Technology (*n* = 35), Peking University (*n* = 34), Tufts University (*n* = 33), Fourth Military Medical University (*n* = 31), University of Chinese Academy of Sciences (*n* = 28), and Tsinghua University (*n* = 26). The VOSviewer software was used to generate the institution cooperation network, and the threshold value of the minimum number of documents of an institution was set to 15, and the minimum number of citations of an institution was set to 500.22 of the 1,652 institutions were identified. In the recent 20 years, Chinese Academy of Sciences has cooperated with the majority of most influential academic institutions to carry out biomaterials research in osteogenesis (Figure 4).

**TABLE 2:** The top 10 most productive institutions in biomaterials research in osteogenesis.

Rank	Institution	Records	Percentage (*n*/1,523) %
1	Chinese academy of sciences	125	8.207
2	Shanghai jiao tong university	110	7.223
3	Sichuan university	75	4.924
4	Zhejiang university	37	2.429
5	Queensland university of technology	35	2.298
6	Peking university	34	2.232
7	Tufts university	33	2.167
8	Fourth military medical university	31	2.035
9	University of chinese academy of sciences	28	1.838
10	Tsinghua university	26	1.707

### Contribution of journals

This study systematically analyzed journal contributions with journal characteristics from journal titles, number of articles, total number of citations, average number of citations, IF (2020), quartile in category (2020), and h-index. Table 3 listed the 10 most productive journals of biomaterials research in osteogenesis. A total of 503 papers were published, accounting for 33.03% of the total number of papers published. Acta Biomaterialia (*n* = 101), Biomaterials (*n* = 80), and Journal of Biomedical Materials Research Part A (*n* = 64) ranked top three in the number of papers published in biomaterials research in osteogenesis (Table 3). Acta Biomaterialia and Biomaterials were the two most frequently cited journals, with a total citation frequency of 388 and 713, respectively. In addition, the above two journals were also the two journals with the highest average citation frequency, which were 3.84 and 8.91 respectively. Biomaterialia, ACS Applied Materials & Interfaces, and Acta Biomaterialia had the highest IFs in 2020, which were 12.479, 9.229, and 8.947, respectively. Biomaterials had the highest h-index value of 65. According to JCR 2020 standard, in the top 10 journals, Acta Biomaterialia, Biomaterials, Materials Science & Engineering C-Materials for Biological Applications, ACS Applied Materials & Interfaces, International Journal of Molecular Sciences, and Biomaterials Science are divided into Q1 (Table 3). The top 10 high-cited papers were listed in Table 4.

**TABLE 3:** The top 10 most active journals that published articles in biomaterials research in osteogenesis.

Rank	Journal title	Article counts	Total number of citations	Average number of citations	IF (2020)	Quartile in category (2020)	h-index
1	Acta Biomaterialia	101	388	3.84	8.947	Q1	56
2	Biomaterials	80	713	8.91	12.479	Q1	65
3	Journal of Biomedical Materials Research Part A	64	127	1.98	4.396	Q2	27
4	Journal of Materials Chemistry B	53	99	1.87	6.331	Q2	45
5	Materials Science & Engineering C - Materials for Biological Applications	52	54	1.04	7.328	Q1	61
6	ACS Applied Materials & Interfaces	48	102	2.13	9.229	Q1	63
7	ACS Biomaterials Science & Engineering	28	19	0.68	4.749	Q2	34
8	Tissue Engineering Part A	26	33	1.27	3.845	Q3	17
9	International Journal of Molecular Sciences	26	12	0.46	5.924	Q1	32
10	Biomaterials Science	25	58	2.32	6.843	Q1	37

**TABLE 4:** The top 10 high-cited papers in biomaterials research in osteogenesis during 2000–2021.

Rank	Title	Authors	Year	Journal	Total citations
1	Porosity of 3D biomaterial scaffolds and osteogenesis	Karageorgiou, V. et al.	2005	Biomaterials	4,326
2	A review of the biological response to ionic dissolution products from bioactive glasses and glass-ceramics	Hoppe, A. et al.	2011	Biomaterials	1,618
3	Properties of osteoconductive biomaterials: Calcium phosphates	LeGeros, RZ. et al.	2002	Clinical orthopaedics and related research	1,345
4	Degradation-mediated cellular traction directs stem cell fate in covalently crosslinked three-dimensional hydrogels	Khetan, S. et al.	2013	Nature materials	779
5	State of the art and future directions of scaffold-based bone engineering from a biomaterials perspective	Hutmacher, DW. et al.	2007	Journal of tissue engineering and regenerative medicine	693
6	The inflammatory responses to silk films *in vitro* and *in vivo*	Meinel, L. et al.	2005	Biomaterials	634
7	Bioactive Glass and Glass-Ceramic Scaffolds for Bone Tissue Engineering	Gerhardt, LC. et al.	2010	Materials	629
8	Influence of engineered titania nanotubular surfaces on bone cells	Popat, KC. et al.	2007	Biomaterials	528
9	Bone augmentation techniques	McAllister, BS. et al.	2007	Journal of periodontology	470
10	Bone tissue engineering using human mesenchymal stem cells: Effects of scaffold material and medium flow	Meinel, L. et al.	2004	Annals of Biomedical engineering	433

### Contribution of authors

**FIGURE 5:**
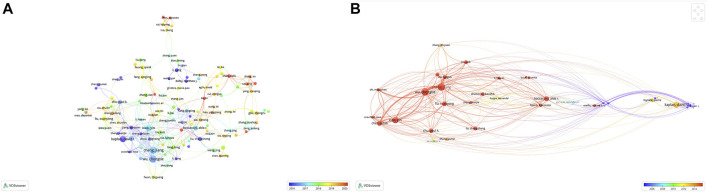
The distribution of authors in biomaterials research in osteogenesis. **(A)** Author co-authorship overlay visualization map. The color of each circle corresponds to the average publication year of the author, the size of a circle is proportional to the number of literatures published by the author, and the thickness of the connecting line indicates the cooperation frequency. **(B)** Author co-citation overlay visualization map. The color of each circle corresponds to the average publication year of the author, the size of a circle is proportional to the total number of citations of the author, and the thickness of the connecting line indicates the strength of the co-citation link.

The top 10 most productive authors in the field of biomaterials research in osteogenesis were listed in Table 5. Among them, the top three authors were Wu CT (*n* = 36) from Shanghai Institute of Ceramics, Chinese Academy of Sciences in China, Chang J (*n* = 36) from Shanghai Institute of Ceramics, Chinese Academy of Sciences in China, and Kaplan DL (*n* = 30) from Department of Biomedical Engineering, Tufts University in United States. In addition, Wu CT, Xiao Y, and Chang J were the top three authors with the highest total number of citations, which were 430, 370 and 354 times respectively (Table 5). A co-authorship overlay visualization map was generated using VOSviewer software and the threshold of the minimum number of documents for an author was set to 5. Finally, 116 authors meeting the threshold were identified. Among them, Wu CT, Chang J, and Xiao Y worked closely together (Figure 5A). A co-citation overlay visualization map was generated using VOSviewer software and the threshold of the minimum number of citations for an author was set to 500. Finally, 49 authors meeting the threshold were identified. Among them, Wu CT, Chang J, and Kaplan, DL made significant contributions in the field of biomaterials research in osteogenesis (Figure 5B and Table 5).

**TABLE 5:** The top 10 most productive authors in biomaterials research in osteogenesis.

Rank	Author	Article counts	Total number of citations	Average number of citations	First author counts	First author citation counts	Corresponding author counts	Corresponding author citation counts
1	Wu, CT	36	430	11.94	3	61	11	83
2	Chang, J	36	354	9.83	0	0	7	78
3	Kaplan, DL	30	118	3.93	0	0	21	94
4	Wang, Y	29	25	0.86	3	4	2	7
5	Xiao, Y	27	370	13.7	1	0	11	208
6	Zhang, Y	25	17	0.68	3	3	3	2
7	Zhang, J	21	43	2.05	4	29	1	0
8	Liu, Y	21	76	3.62	1	2	2	12
9	Liu, L	17	27	1.59	4	5	1	0
10	Boccaccini, AR	17	101	5.94	0	0	6	82

### Analysis of research hotspots

**FIGURE 6:**
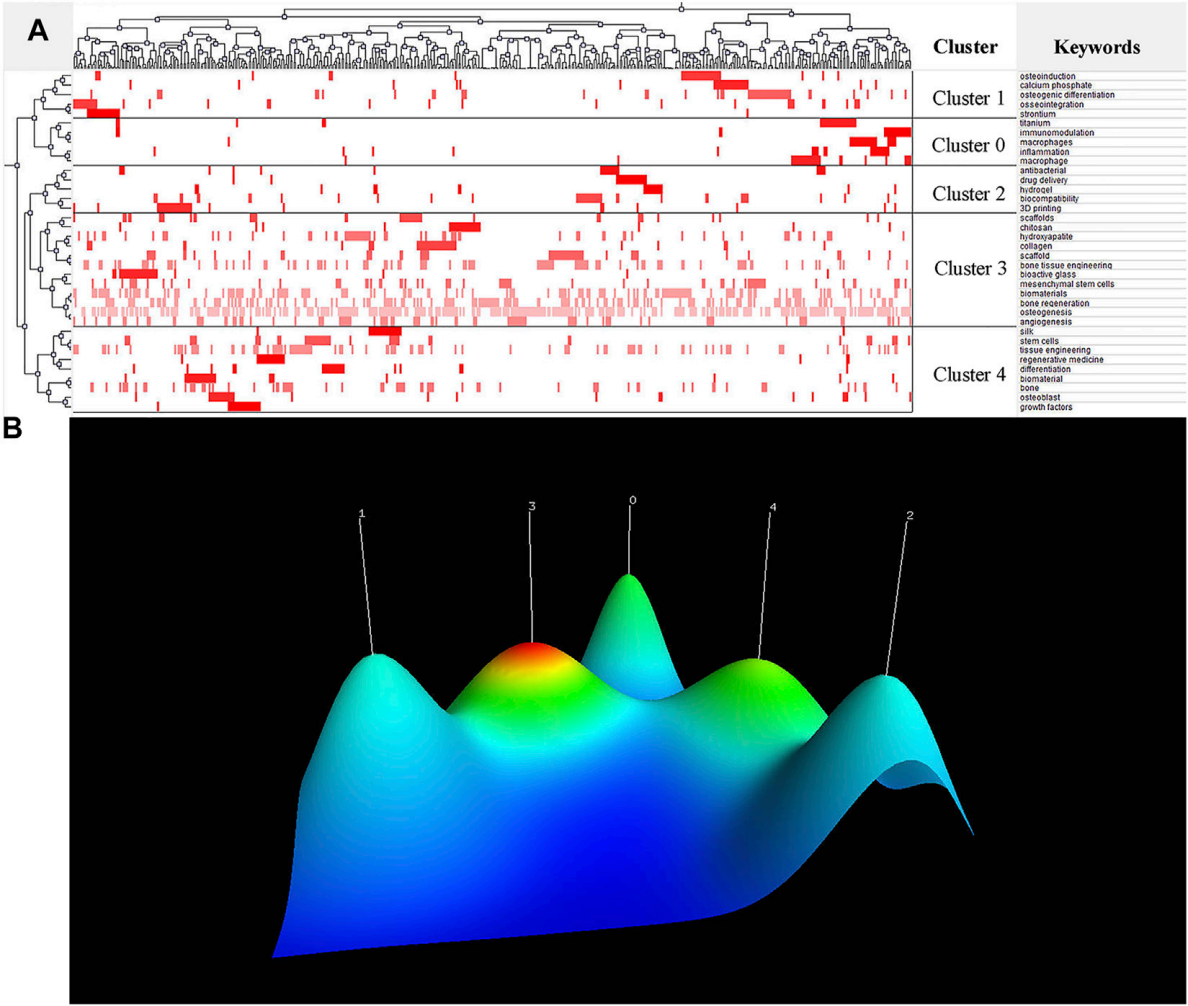
Research hotspots in the field of biomaterials research in osteogenesis. **(A)** Visualized matrix of biclustering of highly frequent keywords in the field of biomaterials research in osteogenesis. Color of each blot represented the frequency of occurrence of keywords in all literatures. **(B)** Mountain visualization of biclustering of highly frequent keywords in the field of biomaterials research in osteogenesis. The height and color of the mountain are proportional to internal similarity and standard deviation of cluster. Blue: high deviation, Red: low deviation.

With more than 20 occurrences, the 36 most frequent keywords were extracted from the included publications and shown in Table 6. The Gcluto double-clustering analysis was used to sort the five clusters. Matrix graph and volcano graph were used to visualize the relationship between publications and high-frequency keywords (Figure 6). The matrix graph was shown in Figure 6A, where column labels represented papers and row labels represented keywords. To combine similar rows into a single cluster, the rows of the initial matrix graph were reset, and each cluster was split by horizontal lines. In the matrix graph, the upper dendrogram represented paper association, and the left represented high-frequency keyword association. The results of the volcano graph in Figure 6B directly characterized the data as five different mountains representing five different clusters numbered from 0 to 4.

**TABLE 6:** Keywords of biomaterials research hotspots in osteogenesis.

Rank	Keywords	Frequency	Percentage (%)
1	osteogenesis	399	5.9490
2	bone regeneration	177	2.6390
3	biomaterials	162	2.4154
4	bone tissue engineering	113	1.6848
5	tissue engineering	110	1.6401
6	angiogenesis	102	1.5208
7	hydroxyapatite	74	1.1033
8	bone	73	1.0884
9	mesenchymal stem cells	66	0.9840
10	scaffold	55	0.8200
11	osteogenic differentiation	51	0.7604
12	stem cells	46	0.6859
13	scaffolds	44	0.6560
14	biocompatibility	40	0.5964
15	collagen	38	0.5666
16	osseointegration	35	0.5218
17	osteoinduction	33	0.4920
18	osteoblast	30	0.4473
19	3D printing	30	0.4473
20	bioactive glass	29	0.4324
21	calcium phosphate	29	0.4324
22	titanium	28	0.4175
23	macrophage	27	0.4026
24	biomaterial	26	0.3877
25	chitosan	25	0.3727
26	antibacterial	24	0.3578
27	inflammation	23	0.3429
28	differentiation	23	0.3429
29	immunomodulation	22	0.3280
30	macrophages	22	0.3280
31	regenerative medicine	21	0.3131
32	silk	21	0.3131
33	drug delivery	20	0.2982
34	hydrogel	20	0.2982
35	growth factors	20	0.2982
36	strontium	20	0.2982

The above 36 high-frequency keywords were divided into 5 clusters (Figure 6A). All representative papers involved in each cluster were excavated to further summarize research hotspots in the field of biomaterials research in osteogenesis. Finally, BICOMB and Gcluto software were used to identify five research hotspots:

Cluster 0: The immunomodulatory role of biomaterial-related inflammatory.

Cluster 1: Mechanisms of osteogenesis in biomaterials.

Cluster 2: 3D printing and clinical application of biomaterials.

Cluster 3: Bone tissue engineering for biomaterial osteogenesis.

Cluster 4: Regenerative medicine for biomaterial osteogenesis.

**FIGURE 7:**
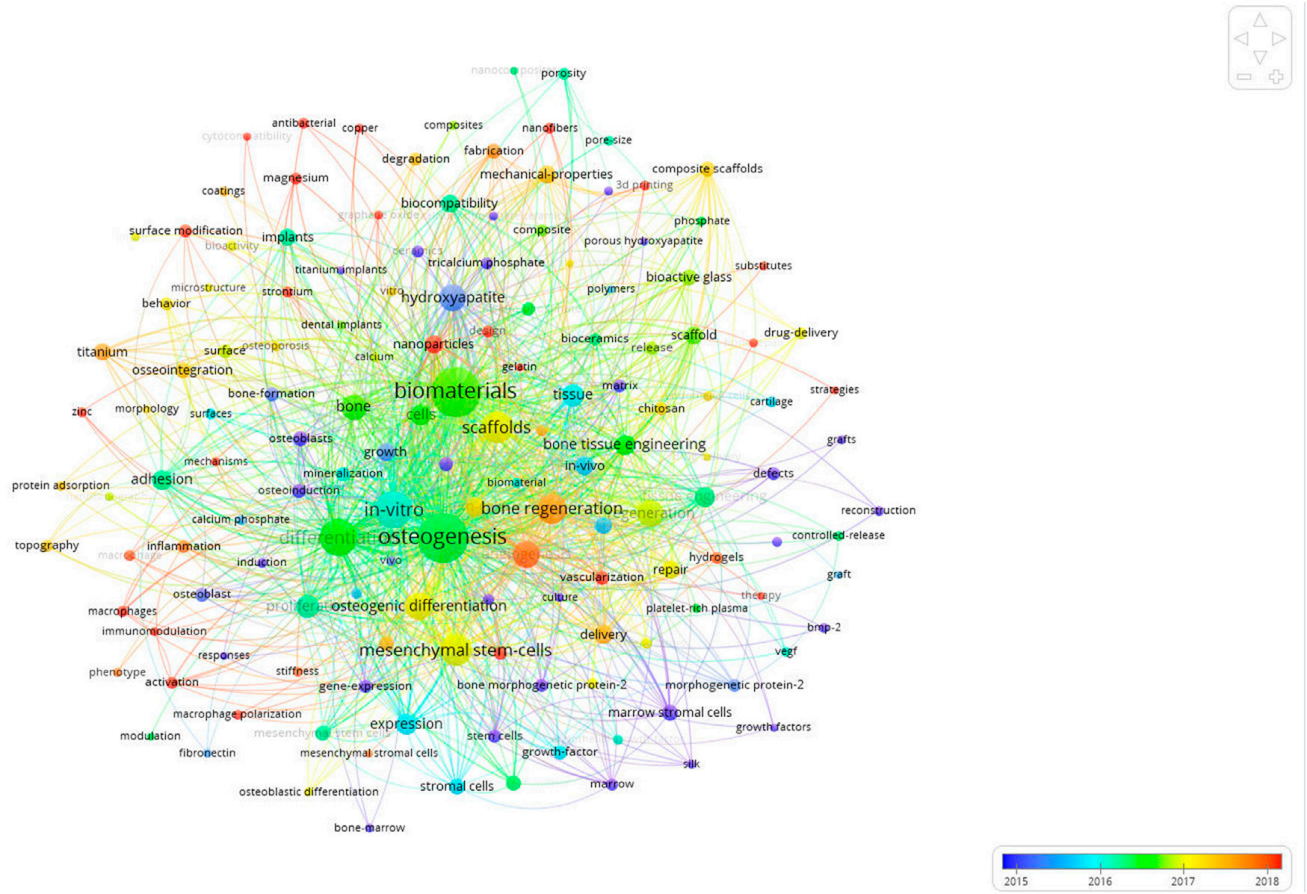
Co-occurrence overlay visualization map of keywords. The color of each circle corresponds to the average publication year. The size of a circle is proportional to the frequency of occurrence of the keyword, and the thickness of the connecting line indicates the strength of the keywords co-occurrence link.

In order to analyze the changes of research hotspots in a period of time, VOSviewer software was used to generate the co-occurrence overlay visualization map of keywords, and the results showed that the keywords “bone regeneration”, “mesenchymal stem cells”, “vascularization”, “surface modification”, and “nanoparticles” have appeared frequently in the last 5 years (Figure 7).

## Discussion

Bibliometric analysis can intuitively visualize the structures of literature data. By presenting visual results, bibliometric analysis can help researchers understand and master the research hotspots and research frontiers in their research fields (Yin M. et al., 2022; Chu et al., 2022). In addition, bibliometric analysis can systematically analyze the information of papers, authors, institutions, and countries or regions, so as to find the papers, authors, and institutions with the most academic value and influence (Glanville et al., 2011; Ramos et al., 2019; Koo, 2021). In this study, we conducted a systematic bibliometric analysis of the global academic publications of the field of biomaterials research in osteogenesis from 2000 to 2021.

The number of publications can reflect the academic productivity and development of the research fields in which the researchers work (Joshi, 2014; García-Villar and García-Santos, 2021). A total of 1,523 papers were included in this study, including 261 papers from 2021 (Figures 1, 2). According to the results of our study, in the 20 years from 2000 to 2021, the number of publications published in the field of biomaterials research in osteogenesis showed a general trend of gradual growth. This trend showed that biomaterials research in osteogenesis would still be a hot research field, and more and more scholars would participate in the field of biomaterials research in osteogenesis. In addition, scholars also pay more attention to the exploration of novel biomaterials.

The number of publications in a certain research field is an important indicator to measure the level of scientific research of an institution, country or region (Joshi, 2014; Koo, 2021). The results of our study showed that China and the United States were the two countries with the largest number of publications published in the field of biomaterials research in osteogenesis (Table 1 and Figure 3A). This highlighted the academic influence of China and the United States in the field of biomaterials research in osteogenesis. In addition, we also noted that Germany had also made a significant contribution in the field of biomaterials research in osteogenesis in the last 20 years. International cooperation is now an important way to realize innovation and solve existing scientific research conundrums (Dara et al., 2017). From 2000 to 2021, many countries or regions in the world had carried out relevant cooperative studies in the field of biomaterials research in osteogenesis. In addition, the results of our study showed that China had the most frequent international cooperative research (Figure 3B). Chinese Academy of Sciences was regarded as the institution with the most academic productivity in the past 20 years (Table 2), and had cooperated and communicated with most influential scientific research institutions in the field of biomaterials research in osteogenesis, including Shanghai Jiaotong University, Sichuan University, Zhejiang University, and Queensland University of Technology (Figure 4). The above results show that countries or regions and scientific research institutions with high academic level tend to cooperate in research, indicating that international cooperative research in the field of biomaterials research in osteogenesis will still be the research trend in the near future.

The journal related metrics data obtained by bibliometric analysis can provide reliable support for scholars to search literatures or publish articles (Chiang et al., 2018; Brandt et al., 2019). Most of the top 10 journals that published papers on the field of biomaterials research in osteogenesis belong to biomaterials and related fields. The results of our study showed that Acta Biomaterialia published the most papers in the field of biomaterials research in osteogenesis, while the total number of citations of Biomaterialia were the highest (Table 3). In addition, the results of our study showed that the most frequently cited paper is Porosity of 3D Biomaterial Scaffolds and Osteogenesis written by Vassilis Karageorgiou and David Kaplan from Department of Chemical and Biological Engineering, Tufts University (Table 4). The above results indicate that these most influential journals and highly cited papers can provide academic inspiration and guidance for scholars in the field of biomaterials research in osteogenesis. Biomaterials research in osteogenesis plays an important role in biomaterials and related fields.

Based on the Web of Science Core Collection database, Wu CT and Chang J published the most papers related to biomaterials research in osteogenesis. While, the scholar with the largest total number of citations was Wu CT (Table 5). In addition, it can be seen from the co-authorship overlay visualization map and the co-citation overlay visualization map that Wu CT, Chang J, Xiao Y, and Kaplan, DL published a considerable number of highly cited papers (Figures 5A,B). The authors mentioned above can be regarded as the leaders in the field of biomaterials research in osteogenesis.

Due to the heterogeneity of the field of biomaterials research in osteogenesis, we divided the keywords in our study into 5 clusters through double-clustering analysis (Figure 6). Cluster 0 was associated with the immunomodulatory role of biomaterial-related inflammatory. Biomaterials are foreign matters to the human body and will cause a series of immune reactions after implantation (Masui et al., 2005; De Avila et al., 2020). Inflammation caused by biomaterials is generally not conducive to the long-term existence of biomaterials in the host, so the focus is on minimizing inflammation. In this inflammatory responses, macrophages may derive from peripheral blood monocytes and become activated, which leads to a range of phenotypes including pro-inflammatory (M1-like), or anti-inflammatory and tissue repair (M2-like) macrophages (Razzi et al., 2020). Recent studies have shown that biomaterials can induce different immune responses (Franz et al., 2011; Brown et al., 2012; Shayan et al., 2018) and that these responses may stimulate subsequent osteogenesis (Li B. et al., 2018; Bai et al., 2018; Sadowska et al., 2018). Cluster one was associated with mechanisms of osteogenesis in biomaterials. Bone is a complex dynamic system in which different biological processes and structural features play complementary roles in osteogenesis (Clarke, 2008). After the biomaterial is implanted into the host, the surface of the material is in direct contact with the bone tissue, so the surface characteristics of the material have a great influence on the osteogenesis. Increasing the surface roughness of biomaterials can increase the contact area between biomaterials and bone tissue. *In vitro*, rough biomaterial surfaces could promote osteoblast differentiation and mineralization, leading to osteogenesis (Anselme and Bigerelle, 2005; Novaes et al., 2010). Osteoblasts cultured on rough surfaces increased the production of alkaline phosphatase and osteocalcin, which were cellular markers of osteoblast differentiation (Schwartz et al., 1999). Cluster two was associated with 3D printing and clinical application of biomaterials. Porous biomaterial scaffolds made by 3D printing can reduce their elastic modulus and match that of bone tissue, thus facilitating osteogenesis (Li et al., 2016b). More importantly, 3D printing can produce irregularly shaped or patient-specific biomaterial scaffolds to meet practical clinical needs (Xiu et al., 2016). In addition, 3D-printed biomaterial scaffolds contain internal lattice structures that can accommodate newly formed bone tissue, enabling good bone-implant integration (Wang et al., 2014; Shah et al., 2016). Cluster three was associated with bone tissue engineering for biomaterial osteogenesis. Bone tissue engineering is a Frontier interdisciplinary discipline in the field of life science in the 21st century, which consists of bioengineering, cell transplantation, and materials science, aiming at constructing biological substitutes for bone injury repair (Qi et al., 2021). Bone tissue-engineered biomaterial scaffolds provide a three-dimensional space for cell proliferation, tissue growth, and vascularization, thereby facilitating osteogenesis. Good bone tissue-engineered biomaterial scaffolds have osteogenesis, osteoconductivity, osteointegration, and osteoinductivity, thus simulating new bone formation (Velasco et al., 2015). The porosity and pore size of biomaterial scaffolds are important factors to regulate the degradation and mechanical properties of scaffolds, thus promoting cell differentiation and new tissue formation (Wang et al., 2015). At present, natural biomaterials, synthetic biomaterials, and metal materials are widely used in bone tissue engineering (García-Gareta et al., 2015). Cluster four was associated with regenerative medicine for biomaterial osteogenesis. Bone is a dynamic, vascularized tissue with a strong regenerative capacity. Chitosan scaffolds can promote the proliferation and attachment of osteoblasts and mesenchymal stem cells, contributing to osteogenesis and thus promoting bone regeneration (LogithKumar et al., 2016). Phosphorylated and sulfate chitosan can promote mineralization and vascularization in the presence of bone morphogenetic protein 2, which can provide a suitable platform for bone regeneration (Pan et al., 2018). Therefore, chitosan scaffolds play an important role in the field of regenerative medicine for biomaterial osteogenesis. Although cluster 3 (bone tissue engineering for biomaterial osteogenesis) and cluster 4 (regenerative medicine for biomaterial osteogenesis) are similar groups, there are still some differences. Regenerative medicine for biomaterial osteogenesis, for example, places more emphasis on the role of biomaterials in bone regeneration. However, compared with regenerative medicine for biomaterial osteogenesis, bone tissue engineering for biomaterial osteogenesis focuses not only on the application of biomaterials in promoting bone growth, but also on the biomechanical and supporting effects of biomaterials *in vivo*. Therefore, the above two have both difference and connection. Bone tissue engineering and regenerative medicine are important research directions in the field of biomaterials research in osteogenesis in the future.

Keyword co-occurrence overlay visualization analysis is a widely accepted method to identify research hotspots and predict research trends (Chen, 2004). The results of our study showed that the keywords such as “bone regeneration”, “mesenchymal stem cells”, “vascularization”, “surface modification”, and “nanoparticles”, in recent 5 years, appeared frequently (Figure 7), indicating that mechanisms of osteogenesis in biomaterials, bone tissue engineering for biomaterial osteogenesis, and regenerative medicine for biomaterial osteogenesis will still be the research hotspots in the future for many years. The skeletal system contains a holistic system of mesenchymal stem cells, osteoprogenitor cells, and osteoblasts to maintain lifelong bone formation. Osteogenesis is essential for the homeostatic renewal of bone and regenerative fracture healing. There is a coupling relationship between the growth of blood vascular and osteogenesis in bone. A study has demonstrated the existence of a novel subtype of capillaries in the mouse skeletal system with unique morphological, molecular and functional properties (Kusumbe et al., 2014). These vessels are located in specific locations to mediate the growth of the bone vessels, generate different metabolic and molecular microenvironments, maintain perivascular osteoprogenitor cells and couple angiogenesis to osteogenesis. This provides an inspiration for the research direction of biomaterials acting on vascularization to promote osteogenesis. Takeuchi et al. (2019) found that mesenchymal stem cells derived exosomes promoted bone regeneration and enhanced vascularization at early stage. This provides enlightenment for the research direction of biomaterials acting on mesenchymal stem cells to promote osteogenesis. Biomaterials with suitable surface modification strategies have made important contributions to the rapid development of bone tissue engineering. Surface modification of biomaterials focuses on enhancing the bioactivity and osteoinductivity of biomaterials on the basis of reducing the intrinsic elastic modulus of biomaterials to achieve osteogenesis. Carbonated hydroxyapatite bioceramic coatings have good surface bioactivity and biocompatibility, as well as better wetting, thus improving protein adhesion and enhancing biological cascade events of bone marrow mesenchymal stem cells, including cell adhesion, proliferation, osteogenic differentiation, and especially the production of proangiogenic growth factor (Li S. et al., 2018). In addition, micro-arc oxidation is a novel and effective method to prepare nanoporous coatings with high bioactivity and osteogenesis. Li et al. (2020) added biological magnesium (Mg2+) to a coating by micro-arc oxidation and developed the nanoporous coatings with excellent osteogenesis *in vitro*. Surface modification of biomaterials makes it possible for some materials with poor bioactivity and osteoinductivity to be applied in the field of bone regeneration. Polyetheretherketone (PEEK) is a promising polymeric material for orthopedic implants due to its suitable mechanical properties that well match natural cortical bone tissue. However, the inert biological properties of PEEK limit its clinical application. One study introduced eucommia ulmoides polysaccharides to the surface of PEEK *via* polydopamine-based coating and form a bioactive PEEK material, thereby enhancing its osteointegration with bone tissue (Mengdi et al., 2022). The introduction of nanoparticles into bone tissue engineering strategies is beneficial to osteogenesis and the regeneration of large bone defects. Li et al. (2020) designed zeolitic imidazolate framework-8 nanoparticle modified catechol-chitosan multifunctional hydrogels are biocompatible and enhance paracrine of vascular endothelial growth factor in bone marrow mesenchymal stem cells, ensuring the reconstruction of blood supply to bone defects. In addition, zeolitic imidazolate framework-8 nanoparticless released by hydrogel can also up-regulate the production and secretion of alkaline phosphatase, collagen 1, and osteocalcin, and promote the osteogenic differentiation of bone marrow mesenchymal stem cells. In addition, Ghosh et al. (2022) prepared hybrid nanocomposites by using graphene nanoribbons and nanoparticles of hydroxyapatite, which could potentially improve osteogenesis. A study showed that the silk fibroin/nano-hydroxyapatite/hyaluronic acid composite scaffolds had excellent cell proliferation and osteogenic differentiation ability (Wang et al., 2021). This further indicates that the nanostructure has a good application prospect in the field of biomaterial osteogenesis. Our study also shows that many scholars are still making efforts to explore the field of biomaterials research in osteogenesis, in order to produce biomaterials with excellent osteogenesis performance so as to be applied in clinical practice as soon as possible in the future.

The literature data about biomaterials research in osteogenesis were all obtained from the Web of Science Core Collection database. After that, bibliometric analysis and visualized analysis were used to systematically analyze the research status of the field of biomaterials research in osteogenesis, making this study relatively comprehensive and objective. However, there are still some limitations in this study. Papers published before 2000 and in 2022 were not included in this study. In addition, non-English language papers were not included in this study. Therefore, subsequent studies will include papers published before 2000 and recently as well as papers in non-English languages to supplement and refine this study.

## Conclusion

To sum up, in the 20 years from 2000 to 2021, the number of annual papers about biomaterials research in osteogenesis showed a trend of continuous growth. China is the leading country in the field of biomaterials research in osteogenesis, and Chinese Academy of Sciences has also achieved important relevant research results. It plays a certain role in promoting the development of biomaterials research in osteogenesis. In addition, Wu CT, Chang J, Xiao Y, and Kaplan, DL made important contributions in the field of biomaterials research in osteogenesis. Research hotspots analysis showed that mechanisms of osteogenesis in biomaterials, bone tissue engineering for biomaterial osteogenesis, and regenerative medicine for biomaterial osteogenesis will still be the research hotspots in the future. International cooperation is increasingly favored by various countries or regions and scientific research institutions, which is very important for the expansion and deepening of biomaterials research in osteogenesis. The results of this study provide a research basis and a new Frontier for biomaterials research in osteogenesis in the future.

## Data Availability

The raw data supporting the conclusions of this article will be made available by the authors, without undue reservation.

## References

[B1] Abdullah M. R. Goharian A. Abdul Kadir M. R. Wahit M. U. (2015). Biomechanical and bioactivity concepts of polyetheretherketone composites for use in orthopedic implants-a review. J. Biomed. Mat. Res. A 103, 3689–3702. 10.1002/jbm.a.35480 25856801

[B2] Albrektsson T. Brånemark P. I. Hansson H. A. Lindström J. (1981). Osseointegrated titanium implants. Requirements for ensuring a long-lasting, direct bone-to-implant anchorage in man. Acta Orthop. Scand. 52, 155–170. 10.3109/17453678108991776 7246093

[B3] Albrektsson T. Johansson C. (2001). Osteoinduction, osteoconduction and osseointegration. Eur. Spine J. 2 (10), S96–S101. 10.1007/s005860100282 PMC3611551 11716023

[B4] Anselme K. Bigerelle M. (2005). Topography effects of pure titanium substrates on human osteoblast long-term adhesion. Acta Biomater. 1, 211–222. 10.1016/j.actbio.2004.11.009 16701798

[B5] Avila J. D. Stenberg K. Bose S. Bandyopadhyay A. (2021). Hydroxyapatite reinforced Ti6Al4V composites for load-bearing implants. Acta Biomater. 123, 379–392. 10.1016/j.actbio.2020.12.060 33450413 PMC7923959

[B6] Bai L. Du Z. Du J. Yao W. Zhang J. Weng Z. (2018). A multifaceted coating on titanium dictates osteoimmunomodulation and osteo/angio-genesis towards ameliorative osseointegration. Biomaterials 162, 154–169. 10.1016/j.biomaterials.2018.02.010 29454274

[B7] Brandt J. S. Hadaya O. Schuster M. Rosen T. Sauer M. V. Ananth C. V. (2019). A bibliometric analysis of top-cited journal articles in obstetrics and gynecology. JAMA Netw. Open 2, e1918007. 10.1001/jamanetworkopen.2019.18007 31860106 PMC6991228

[B8] Brown B. N. Ratner B. D. Goodman S. B. Amar S. Badylak S. F. (2012). Macrophage polarization: An opportunity for improved outcomes in biomaterials and regenerative medicine. Biomaterials 33, 3792–3802. 10.1016/j.biomaterials.2012.02.034 22386919 PMC3727238

[B9] Chen C. (2004). Searching for intellectual turning points: Progressive knowledge domain visualization. Proc. Natl. Acad. Sci. U. S. A. 1 (101), 5303–5310. 10.1073/pnas.0307513100 PMC387312 14724295

[B10] Chen X. Lian T. Zhang B. Du Y. Du K. Xiang N. (2021). Design and mechanical compatibility of nylon bionic cancellous bone fabricated by selective laser sintering. Mater. (Basel) 14, 1965. 10.3390/ma14081965 PMC8070912 33919911

[B11] Chiang H. S. Huang R. Y. Weng P. W. Mau L. P. Tsai Y. C. Chung M. P. (2018). Prominence of scientific publications towards peri-implant complications in implantology: A bibliometric analysis using the H-classics method. J. Oral Rehabil. 45, 240–249. 10.1111/joor.12606 29314191

[B12] Chou W. C. Wang R. C. Liu C. Yang C. Y. Lee T. M. (2017). Surface modification of direct-current and radio-frequency oxygen plasma treatments enhance cell biocompatibility. Mater. (Basel) 10, 1223. 10.3390/ma10111223 PMC5706170 29068417

[B13] Chu P. L. Wang T. Zheng J. L. Xu C. Q. Yan Y. J. Ma Q. S. (2022). Global and current research trends of unilateral biportal endoscopy/biportal endoscopic spinal surgery in the treatment of lumbar degenerative diseases: A bibliometric and visualization study. Orthop. Surg. 14, 635–643. 10.1111/os.13216 35293686 PMC9002063

[B14] Clarke B. (2008). Normal bone anatomy and physiology. Clin. J. Am. Soc. Nephrol. 3 (3), S131–S139. 10.2215/cjn.04151206 18988698 PMC3152283

[B15] Cooper I. D. (2015). Bibliometrics basics. J. Med. Libr. Assoc. 103, 217–218. 10.3163/1536-5050.103.4.013 26512226 PMC4613387

[B16] Dara M. Sulis G. Centis R. D'ambrosio L. De Vries G. Douglas P. (2017). Cross-border collaboration for improved tuberculosis prevention and care: Policies, tools and experiences. Int. J. Tuberc. lung Dis. 21, 727–736. 10.5588/ijtld.16.0940 28633696

[B17] De Avila E. D. Van Oirschot B. A. Van Den Beucken J. (2020). Biomaterial-based possibilities for managing peri-implantitis. J. Periodontal Res. 55, 165–173. 10.1111/jre.12707 31638267 PMC7154698

[B18] Deng P. Wang S. Sun X. Qi Y. Ma Z. Pan X. (2022). Global trends in research of gouty arthritis over past decade: A bibliometric analysis. Front. Immunol. 13, 910400. 10.3389/fimmu.2022.910400 35757713 PMC9229989

[B19] Franz S. Rammelt S. Scharnweber D. Simon J. C. (2011). Immune responses to implants - a review of the implications for the design of immunomodulatory biomaterials. Biomaterials 32, 6692–6709. 10.1016/j.biomaterials.2011.05.078 21715002

[B20] Furko M. Bella E. D. Fini M. Balázsi C. (2019). Corrosion and biocompatibility examination of multi-element modified calcium phosphate bioceramic layers. Mater. Sci. Eng. C 95, 381–388. 10.1016/j.msec.2018.01.010 30573262

[B21] García-Gareta E. Coathup M. J. Blunn G. W. (2015). Osteoinduction of bone grafting materials for bone repair and regeneration. Bone 81, 112–121. 10.1016/j.bone.2015.07.007 26163110

[B22] García-Villar C. García-Santos J. M. (2021). Bibliometric indicators to evaluate scientific activity. Radiologia 63, 228–235. 10.1016/j.rx.2021.01.002 33593607

[B23] Ghosh S. Bhagwat T. Kitture R. Thongmee S. Webster T. J. (2022). Synthesis of graphene-hydroxyapatite nanocomposites for potential use in bone tissue engineering. J. Vis. Exp. 185, 63985. 10.3791/63985 35969088

[B24] Glanville J. Kendrick T. Mcnally R. Campbell J. Hobbs F. D. (2011). Research output on primary care in Australia, Canada, Germany, The Netherlands, the United Kingdom, and the United States: Bibliometric analysis. BMJ 342, d1028. 10.1136/bmj.d1028 21385804 PMC3050436

[B25] Harb S. V. Uvida M. C. Trentin A. Oliveira Lobo A. Webster T. J. Pulcinelli S. H. (2020). PMMA-silica nanocomposite coating: Effective corrosion protection and biocompatibility for a Ti6Al4V alloy. Mater. Sci. Eng. C 110, 110713. 10.1016/j.msec.2020.110713 32204025

[B26] Joshi M. A. (2014). Bibliometric indicators for evaluating the quality of scientific publications. J. Contemp. Dent. Pract. 15, 258–262. 10.5005/jp-journals-10024-1525 25095854

[B27] Kaur M. Singh K. (2019). Review on titanium and titanium based alloys as biomaterials for orthopaedic applications. Mater. Sci. Eng. C 102, 844–862. 10.1016/j.msec.2019.04.064 31147056

[B28] Koo M. (2021). Systemic lupus erythematosus research: A bibliometric analysis over a 50-year period. Int. J. Environ. Res. Public Health 18, 7095. 10.3390/ijerph18137095 34281030 PMC8295925

[B29] Kusumbe A. P. Ramasamy S. K. Adams R. H. (2014). Coupling of angiogenesis and osteogenesis by a specific vessel subtype in bone. Nature 507, 323–328. 10.1038/nature13145 24646994 PMC4943525

[B30] Li B. Gao P. Zhang H. Guo Z. Zheng Y. Han Y. (2018a). Osteoimmunomodulation, osseointegration, and *in vivo* mechanical integrity of pure Mg coated with HA nanorod/pore-sealed MgO bilayer. Biomater. Sci. 6, 3202–3218. 10.1039/c8bm00901e 30328849

[B31] Li G. Cao H. Zhang W. Ding X. Yang G. Qiao Y. (2016a). Enhanced osseointegration of hierarchical micro/nanotopographic titanium fabricated by microarc oxidation and electrochemical treatment. ACS Appl. Mat. Interfaces 8, 3840–3852. 10.1021/acsami.5b10633 26789077

[B32] Li G. Wang L. Pan W. Yang F. Jiang W. Wu X. (2016b). *In vitro* and *in vivo* study of additive manufactured porous Ti6Al4V scaffolds for repairing bone defects. Sci. Rep. 6, 34072. 10.1038/srep34072 27667204 PMC5036184

[B33] Li S. Yu W. Zhang W. Zhang G. Yu L. Lu E. (2018b). Evaluation of highly carbonated hydroxyapatite bioceramic implant coatings with hierarchical micro-/nanorod topography optimized for osseointegration. Int. J. Nanomedicine 13, 3643–3659. 10.2147/ijn.s159989 29983560 PMC6027846

[B34] Li X. Wang M. Zhang W. Bai Y. Liu Y. Meng J. (2020). A magnesium-incorporated nanoporous titanium coating for rapid osseointegration. Int. J. Nanomedicine 15, 6593–6603. 10.2147/ijn.s255486 32982220 PMC7490434

[B35] Li Y. Ding Y. Munir K. Lin J. Brandt M. Atrens A. (2019). Novel β-Ti35Zr28Nb alloy scaffolds manufactured using selective laser melting for bone implant applications. Acta Biomater. 87, 273–284. 10.1016/j.actbio.2019.01.051 30690210

[B36] Lin X. Yang S. Lai K. Yang H. Webster T. J. Yang L. (2017). Orthopedic implant biomaterials with both osteogenic and anti-infection capacities and associated *in vivo* evaluation methods. Nanomedicine Nanotechnol. Biol. Med. 13, 123–142. 10.1016/j.nano.2016.08.003 27553074

[B37] Liu Y. Zhu Z. Pei X. Zhang X. Cheng X. Hu S. (2020). ZIF-8-Modified multifunctional bone-adhesive hydrogels promoting angiogenesis and osteogenesis for bone regeneration. ACS Appl. Mat. Interfaces 12, 36978–36995. 10.1021/acsami.0c12090 32814397

[B38] Logithkumar R. Keshavnarayan A. Dhivya S. Chawla A. Saravanan S. Selvamurugan N. (2016). A review of chitosan and its derivatives in bone tissue engineering. Carbohydr. Polym. 151, 172–188. 10.1016/j.carbpol.2016.05.049 27474556

[B39] Man Z. Li T. Zhang L. Yuan L. Wu C. Li P. (2018). E7 peptide-functionalized Ti6Al4V alloy for BMSC enrichment in bone tissue engineering. Am. J. Transl. Res. 10, 2480–2490.doi 30210686 PMC6129526

[B40] Masui T. Sakano S. Hasegawa Y. Warashina H. Ishiguro N. (2005). Expression of inflammatory cytokines, RANKL and OPG induced by titanium, cobalt-chromium and polyethylene particles. Biomaterials 26, 1695–1702. 10.1016/j.biomaterials.2004.05.017 15576143

[B41] Meischel M. Hörmann D. Draxler J. Tschegg E. K. Eichler J. Prohaska T. (2017). Bone-implant degradation and mechanical response of bone surrounding Mg-alloy implants. J. Mech. Behav. Biomed. Mat. 71, 307–313. 10.1016/j.jmbbm.2017.03.025 28390303

[B42] Mengdi Z. Jiayi L. Canfeng L. Guofeng W. Yutong W. Pengzhou H. (2022). Surface modification of polyetheretherketone (PEEK) to enhance osteointegration by grafting strontium Eucommia ulmoides polysaccharides. Int. J. Biol. Macromol. 211, 230–237. 10.1016/j.ijbiomac.2022.05.048 35561859

[B43] Navarro M. Michiardi A. Castaño O. Planell J. A. (2008). Biomaterials in orthopaedics. J. R. Soc. Interface 5, 1137–1158. 10.1098/rsif.2008.0151 18667387 PMC2706047

[B44] Novaes A. B. Jr. De Souza S. L. De Barros R. R. Pereira K. K. Iezzi G. Piattelli A. (2010). Influence of implant surfaces on osseointegration. Braz. Dent. J. 21, 471–481. 10.1590/s0103-64402010000600001 21271036

[B45] Okulov I. V. Joo S. H. Okulov A. V. Volegov A. S. Luthringer B. Willumeit-Römer R. (2020). Surface functionalization of biomedical Ti-6Al-7Nb alloy by liquid metal dealloying. Nanomater. (Basel) 10, 1479. 10.3390/nano10081479 PMC7466585 32731588

[B46] Pan Y. Chen J. Yu Y. Dai K. Wang J. Liu C. (2018). Enhancement of BMP-2-mediated angiogenesis and osteogenesis by 2-N, 6-O-sulfated chitosan in bone regeneration. Biomater. Sci. 6, 431–439. 10.1039/c7bm01006k 29340375

[B47] Qi J. Yu T. Hu B. Wu H. Ouyang H. (2021). Current biomaterial-based bone tissue engineering and translational medicine. Int. J. Mol. Sci. 22, 10233. 10.3390/ijms221910233 34638571 PMC8508818

[B48] Ramos M. B. Koterba E. Rosi Júnior J. Teixeira M. J. Figueiredo E. G. (2019). A bibliometric analysis of the most cited articles in neurocritical care research. Neurocrit. Care 31, 365–372. 10.1007/s12028-019-00731-6 31087256

[B49] Razzi F. Fratila-Apachitei L. E. Fahy N. Bastiaansen-Jenniskens Y. M. Apachitei I. Farrell E. (2020). Immunomodulation of surface biofunctionalized 3D printed porous titanium implants. Biomed. Mat. 15, 035017. 10.1088/1748-605x/ab7763 32069447

[B50] Sadowska J. M. Wei F. Guo J. Guillem-Marti J. Ginebra M. P. Xiao Y. (2018). Effect of nano-structural properties of biomimetic hydroxyapatite on osteoimmunomodulation. Biomaterials 181, 318–332. 10.1016/j.biomaterials.2018.07.058 30098568

[B51] Schwartz Z. Lohmann C. H. Oefinger J. Bonewald L. F. Dean D. D. Boyan B. D. (1999). Implant surface characteristics modulate differentiation behavior of cells in the osteoblastic lineage. Adv. Dent. Res. 13, 38–48. 10.1177/08959374990130011301 11276745

[B52] Shah F. A. Omar O. Suska F. Snis A. Matic A. Emanuelsson L. (2016). Long-term osseointegration of 3D printed CoCr constructs with an interconnected open-pore architecture prepared by electron beam melting. Acta Biomater. 36, 296–309. 10.1016/j.actbio.2016.03.033 27000553

[B53] Shayan M. Padmanabhan J. Morris A. H. Cheung B. Smith R. Schroers J. (2018). Nanopatterned bulk metallic glass-based biomaterials modulate macrophage polarization. Acta Biomater. 75, 427–438. 10.1016/j.actbio.2018.05.051 29859902 PMC6119487

[B54] Takeuchi R. Katagiri W. Endo S. Kobayashi T. (2019). Exosomes from conditioned media of bone marrow-derived mesenchymal stem cells promote bone regeneration by enhancing angiogenesis. PLoS One 14, e0225472. 10.1371/journal.pone.0225472 31751396 PMC6872157

[B55] Van Der Stok J. Van Lieshout E. M. El-Massoudi Y. Van Kralingen G. H. Patka P. (2011). Bone substitutes in The Netherlands - a systematic literature review. Acta Biomater. 7, 739–750. 10.1016/j.actbio.2010.07.035 20688196

[B56] Velasco M. A. Narváez-Tovar C. A. Garzón-Alvarado D. A. (2015). Design, materials, and mechanobiology of biodegradable scaffolds for bone tissue engineering. Biomed. Res. Int. 2015, 1–21. 10.1155/2015/729076 PMC4391163 25883972

[B57] Wang J. Yang M. Zhu Y. Wang L. Tomsia A. P. Mao C. (2014). Phage nanofibers induce vascularized osteogenesis in 3D printed bone scaffolds. Adv. Mat. 26, 4961–4966. 10.1002/adma.201400154 PMC4122615 24711251

[B58] Wang L. Nan X. Hou J. Xia Y. Guo Y. Meng K. (2021). Preparation and biological properties of silk fibroin/nano-hydroxyapatite/hyaluronic acid composite scaffold. Biomed. Mat. 16, 045045. 10.1088/1748-605x/ac08aa 16 34098538

[B59] Wang M. O. Vorwald C. E. Dreher M. L. Mott E. J. Cheng M. H. Cinar A. (2015). Evaluating 3D-printed biomaterials as scaffolds for vascularized bone tissue engineering. Adv. Mat. 27, 138–144. 10.1002/adma.201403943 PMC4404492 25387454

[B60] Wong K. C. (2016). 3D-printed patient-specific applications in orthopedics. Orthop. Res. Rev. 8, 57–66. 10.2147/orr.s99614 30774470 PMC6209352

[B61] Wu C. Ramaswamy Y. Gale D. Yang W. Xiao K. Zhang L. (2008). Novel sphene coatings on Ti-6Al-4V for orthopedic implants using sol-gel method. Acta Biomater. 4, 569–576. 10.1016/j.actbio.2007.11.005 18182336

[B62] Xiu P. Jia Z. Lv J. Yin C. Cheng Y. Zhang K. (2016). Tailored surface treatment of 3D printed porous Ti6Al4V by microarc oxidation for enhanced osseointegration via optimized bone in-growth patterns and interlocked bone/implant interface. ACS Appl. Mat. Interfaces 8, 17964–17975. 10.1021/acsami.6b05893 27341499

[B63] Yin M. C. Wang H. S. Yang X. Xu C. Q. Wang T. Yan Y. J. (2022b). A bibliometric analysis and visualization of current research trends in Chinese medicine for osteosarcoma. Chin. J. Integr. Med. 28, 445–452. 10.1007/s11655-020-3429-4 32876857

[B64] Yin M. Wang H. Sun Y. Xu C. Ye J. Ma J. (2022a). Global trends of researches on lumbar spinal stenosis: A bibliometric and visualization study. Clin. Spine Surg. 35, E259–E266. 10.1097/bsd.0000000000001160 33769984

[B65] Yin M. Xu C. Ma J. Ye J. Mo W. (2021). A bibliometric analysis and visualization of current research trends in the treatment of cervical spondylotic myelopathy. Glob. Spine J. 11, 988–998. 10.1177/2192568220948832 PMC8258815 32869687

[B66] Yuan L. Ding S. Wen C. (2019). Additive manufacturing technology for porous metal implant applications and triple minimal surface structures: A review. Bioact. Mat. 4, 56–70. 10.1016/j.bioactmat.2018.12.003 PMC6305839 30596158

[B67] Zhang R. Zhong S. Zeng L. Li H. Zhao R. Zhang S. (2021). Novel Mg-incorporated micro-arc oxidation coatings for orthopedic implants application. Mater. (Basel) 14, 5710. 10.3390/ma14195710 PMC8510346 34640102

